# Phenotypic and Functional Changes of Endothelial and Smooth Muscle Cells in Thoracic Aortic Aneurysms

**DOI:** 10.1155/2016/3107879

**Published:** 2016-01-19

**Authors:** Anna Malashicheva, Daria Kostina, Aleksandra Kostina, Olga Irtyuga, Irina Voronkina, Larisa Smagina, Elena Ignatieva, Natalia Gavriliuk, Vladimir Uspensky, Olga Moiseeva, Jarle Vaage, Anna Kostareva

**Affiliations:** ^1^Almazov Federal Medical Research Centre, Akkuratova 2, Saint Petersburg 197341, Russia; ^2^Saint Petersburg State University, Universitetskaya Nab. 7/9, Saint Petersburg 199034, Russia; ^3^ITMO University, Institute of Translational Medicine, 49 Kronverksky Prospekt, Saint Petersburg 197101, Russia; ^4^Institute of Cytology, Russian Academy of Sciences, Tikhoretsky Avenue 4, Saint Petersburg 194064, Russia; ^5^Institute of Clinical Medicine, University of Oslo and Oslo University Hospital, Postboks 1171, Blindern, 0318 Oslo, Norway

## Abstract

Thoracic aortic aneurysm develops as a result of complex series of events that alter the cellular structure and the composition of the extracellular matrix of the aortic wall. The purpose of the present work was to study the cellular functions of endothelial and smooth muscle cells from the patients with aneurysms of the thoracic aorta. We studied endothelial and smooth muscle cells from aneurysms in patients with bicuspid aortic valve and with tricuspid aortic valve. The expression of key markers of endothelial (CD31, vWF, and VE-cadherin) and smooth muscle (SMA, SM22*α*, calponin, and vimentin) cells as well extracellular matrix and MMP activity was studied as well as and apoptosis and cell proliferation. Expression of functional markers of endothelial and smooth muscle cells was reduced in patient cells. Cellular proliferation, migration, and synthesis of extracellular matrix proteins are attenuated in the cells of the patients. We show for the first time that aortic endothelial cell phenotype is changed in the thoracic aortic aneurysms compared to normal aortic wall. In conclusion both endothelial and smooth muscle cells from aneurysms of the ascending aorta have downregulated specific cellular markers and altered functional properties, such as growth rate, apoptosis induction, and extracellular matrix synthesis.

## 1. Introduction

Thoracic aortic aneurysms have altered cellular composition and degeneration of the extracellular matrix in the aortic wall. There are several different etiologies of thoracic aortic aneurysm such as monogenic syndromes (Marfan and Loeys-Dietz syndromes), aneurysm associated with bicuspid aortic valves, and idiopathic aneurysms. The pathogenesis of aneurysm formation in the monogenic syndromes has been extensively studied [1], whereas the molecular and cellular mechanisms of the other forms, which constitute the majority of thoracic aortic aneurysms, remain largely unknown.

Most studies of the cell and molecular biology of thoracic aortic aneurysm have used entire wall specimens [2–6]. Consequently, the biology of the different cell types in the aneurysmal wall remains largely unknown. Single studies comparing smooth muscle cells (SMCs) from aneurysm patients with the cells from patients with acute aortic dissection revealed differences in expression of some SMC specific genes. In addition, SMCs from aneurysms were demonstrated to have significantly shorter telomeres, reduced metabolic activity, and impaired proliferation and migration rates [7]. Comparison of SMC derived from either bicuspid aortic valve (BAV) or normal tricuspid aortic valve (TAV) in thoracic aortic aneurysms showed expression difference in several markers including osteopontin and tissue inhibitor of metalloproteinase 3 which may reflect different etiologies of TAV- and BAV-associated aneurysms [5–10]. However all above-mentioned studies did not address the functional properties of endothelial cells. As a consequence, a possible role of the endothelium in the developing of aneurysms is largely unknown, although a recent study suggests that aneurysms at least from BAV patients may be associated with endothelial dysfunction [11].

Endothelial cells have a substantial influence on smooth muscle cell differentiation [12]. Recent studies show that endothelial cells could directly influence smooth muscle cell phenotype [13, 14]. The major role of mature differentiated vascular smooth muscle cells is to maintain blood vessel tone and to regulate blood pressure through constriction or relaxation. This is achieved through the expression of a complement of regulatory and contractile genes that provide the machinery for this response [15]. The differentiated contractile phenotype is largely characterized by expression of coordinately regulated smooth muscle-specific markers that include smooth muscle (SM) *α*-actin (*ACTA2*), and SM22*α* (*TAGLN*) and some other proteins [15].

We sought to investigate if SMCs from aortic tissue of the patients with thoracic aneurysm undergo phenotypic and functional change such as growth, apoptosis, and extracellular matrix synthesis and whether this change is also accompanied by endothelial cell changes in the cells of the patient tissues compared to the cells from healthy tissue. Our data demonstrate downregulation of smooth muscle as well as endothelial cell specific markers in the patient cells and also changes in functional state of both SMC and endothelial cells.

## 2. Materials and Methods

The clinical research protocol was approved by the Local Ethics Committee of the Almazov Federal Medical Research Center and was in accordance with the principle of the Declaration of Helsinki. All patients gave informed consent.

Samples of the aneurysmal wall of the thoracic aorta were harvested during aortic surgery at the Almazov Federal Medical Research Center. Thirty specimens were sampled from patients with BAV (*n* = 17) or TAV (*n* = 13) (Table 1). Patients with connective tissue disorders were excluded. Control aortic specimens were obtained from organ transplant donors (*n* = 11) and all had TAV. Donors were all men with mean age 48 ± 11. All tissues were sampled from the outer curvature of the thoracic aorta.

### 2.1. Primary Cultures

To obtain SMC cultures the cells were isolated as previously described [16]. The cells were used in experiments at passages 2–5. Human aortic endothelial cells (HAEC) were isolated from tissue fragments of patients after surgery for aneurysm corrections. Under sterile conditions tissue fragments were dissected away from the adventitia. After washing in PBS, the tissue fragments were first incubated for 30 min at 37°C in 0.1% collagenase solution (Collagenase, Type 3, Worthington Biochemical Corporation, USA). Then endothelial layer was removed mechanically by scraper, and endothelial cells were washed twice and plated onto fresh 3 cm^2^ culture dish covered with 0.1% gelatin (Sigma) in EGM2 medium (Promocell) and incubated at 37°C. The next day endothelial cells were washed by PBS and culture medium was changed. The cells were used in experiments at passages 2–4.

### 2.2. Cell Migration Assay

Cell migration was determined using a “scratch” wound assay as described previously [17]. SMCs were grown to confluence on 6-well plates; after the cells formed a monolayer, the medium was exchanged for serum free medium containing 10 mM hydroxyurea and 10 ng/mL PDGF_BB growth factor to inhibit proliferation and to stimulate migration and the cell monolayer was scraped with a 200P pipette tip to create a cell-free zone. The number of cells which migrated into the wounded area was counted after six and 24 hours. Experiments were performed in duplicate and then repeated three times.

### 2.3. Apoptosis Assay

For estimation of apoptosis SMCs were seeded at a density of 10 × 10^3^ cells/cm^2^, and 10 × 10^−3^ M hydrogen peroxide (H_2_O_2_) was added to the culture medium 48 hours later. After two hours the cells were removed and labeled with FITC-conjugated annexin V (Sigma). The number of annexin V labelled cells was estimated by flow cytometry using Calibur II (BD).

### 2.4. Reverse Transcription-PCR

Total RNA was extracted from SMCs or endothelial cells using Trizol reagent (Invitrogen) according to the instructions of the manufacturer. Reverse transcription was performed using kits (Eurogen, Russia). Real-time PCR was performed in the LightCycler system with SYBR Green detection (Fermentas) using specific primers. The mRNA levels were normalized to GAPDH or HPRT mRNA. Changes in target genes expression levels were calculated as fold differences using the comparative ΔΔCT method. The primer sequence is available upon request.

### 2.5. Immunoblotting

Proteins were extracted from medial tissues or SMCs. Specimens were homogenized in a lysis buffer (50 mM Tris (pH 8), 150 mM NaCl, 1% Triton X-100, 1% sodium deoxycholate, and 5 mM EDTA), containing protease inhibitors (Roche). Extracts were separated by 10% sodium dodecyl sulfate-polyacrylamide gel electrophoresis (SDS-PAGE). Primary antibodies used are SM22*α* (ab14106, Abcam), SMA, vimentin (M072529, DAKO), beta-actin (ab 6276, Abcam), collagen I, fibrillin, and elastin. Positive bands were quantified by densitometry using a gel documentation system Fusion Fix (Vilber Lourmat) and Fusion-Capt software. Bands were normalized using beta-actin stainings.

### 2.6. Immunocytochemistry

Primary antibodies used are SMA (sc-32251, Santa Cruz), SM22alpha (ab14106, Abcam), vimentin (sc-6260, Santa Cruz), VE-cadherin (MAB938, RandD), von Willebrand factor (ab20435, Abcam), and calponin (ab700, Abacam). Secondary antibodies conjugated with Alexa488 or Alexa546 (Invitrogen) were used. DAPI was used to visualize nuclei. Microphotographs were taken using AxioObserver Microscope (Zeiss) at ×20 magnification with AxioVision software.

### 2.7. Zymography

MMP activity was assayed by a modified gelatin zymography method [18]. Activity and content of MMP-2 and MMP-9 were expressed in QuantiScan arbitrary units.

### 2.8. Statistical Analysis

Values are expressed as means ± SD. Groups were compared using the Mann-Whitney nonparametric test. A value of *P* ≤ 0.05 was considered significant.

## 3. Results

### 3.1. Expression of Smooth Muscle Cell Markers in Smooth Muscle Cells from Aneurysms of the Thoracic Aorta

**Figure 1:**
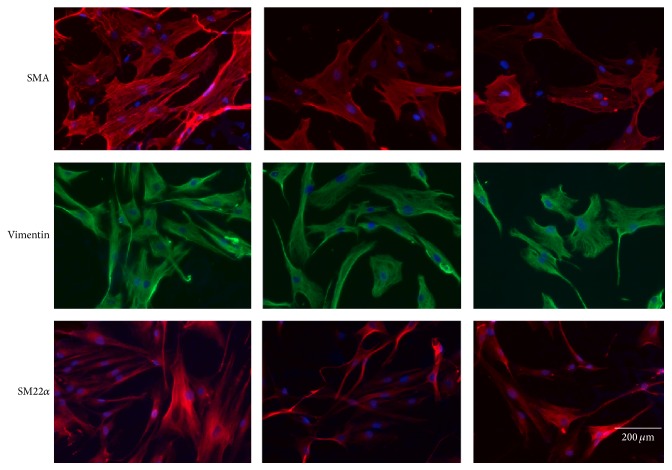
Expression of smooth muscle cell (SMC) markers in SMC from patients with aortic aneurysm with either tricuspid aortic valve (TAV) or bicuspid aortic valve (BAV) and controls (C) determined by immunohistochemical staining of vimentin, *α*-smooth muscle actin (SMA), and SM22*α* (magnification ×20).

SMCs from aneurysms of the thoracic aorta and from control aortas were analyzed regarding the expression of SMC markers like *α*-smooth muscle actin (SMA), vimentin, and SM22*α*.  Figure 1 shows typical immunofluorescent staining of SMC from control aortas and from aneurysms in patients with BAV and TAV. Both TAV- and BAV-derived SMCs appeared to have decreased level of SMA, vimentin, and SM22. However, there were no visible differences between SMC from patients with BAV and TAV.

**Figure 2:**
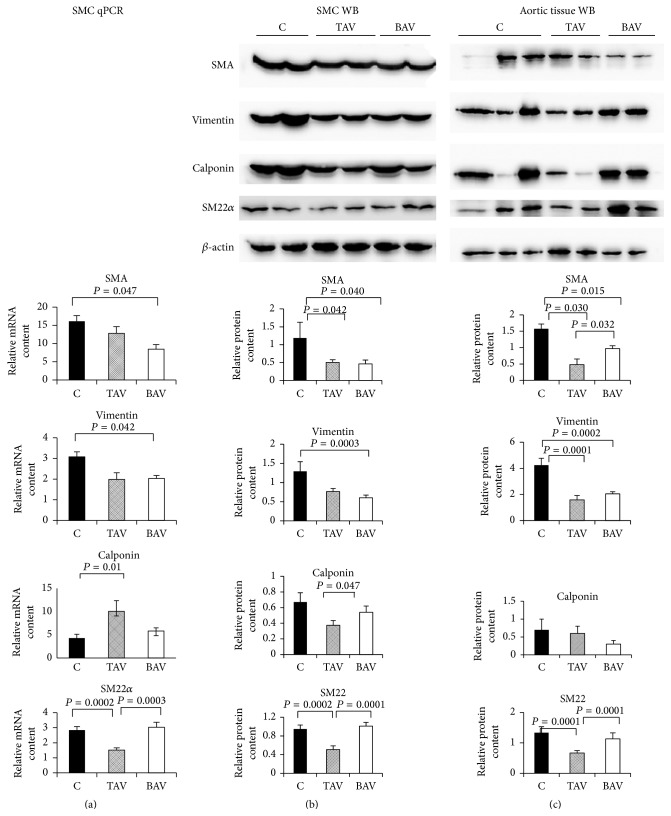
Expression of smooth muscle cell (SMC) markers in SMC from patients with aortic aneurysm with either tricuspid aortic valve (TAV) or bicuspid aortic valve (BAV) and controls (C). mRNA level was determined by qPCR; protein level was determined by Western blot. The diagrams represent the results of densitometry. The bands were normalized by *β*-actin. (a) mRNA level in SMC, C: *n* = 10; TAV: *n* = 13; BAV: *n* = 11. (b) Protein level in SMC, C: *n* = 10; TAV: *n* = 13; BAV: *n* = 11. (c) Protein level in aortic media, C: *n* = 11; TAV: *n* = 13; BAV: *n* = 17.

At both mRNA and protein level expression of SMA and vimentin was reduced both in the BAV- and TAV-derived SMC and in the aortic media (Figure 2). However, although SMA was lower in aortic media of aneurysms from patients with BAV than in controls, it was still higher than in patients with TAV. SM22 expression was decreased only in SMC and aortic media from patients with TAV. In aneurysms from patients with BAV the expression of SMA was higher than in TAV patients both in SMC and in aortic media (Figure 2).

### 3.2. Expression of Endothelial Markers in Endothelial Cells from Aneurysms of the Thoracic Aorta

**Figure 3:**
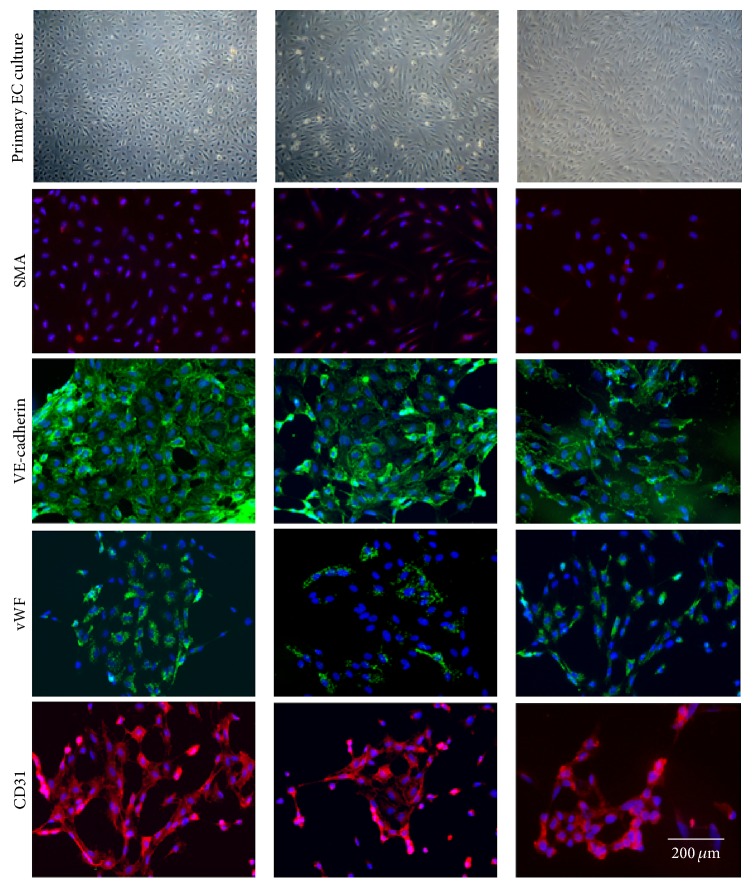
Characterization of the aortic endothelial cells from patients with aortic aneurysm with either tricuspid aortic valve (TAV) or bicuspid aortic valve (BAV) and controls (C). Upper panel represents typical aortic endothelial cell cultures from control and aneurysmal aortas. The SMA staining confirms the lack of medial SMC contamination in the endothelial cell culture. Vascular endothelial-cadherin (VE-cadherin), von Willebrand factor (vWF), and CD31/PECAM staining confirm endothelial nature of the isolated cells.

Figure 3 demonstrates primary cultures of endothelial cells and ICC staining of the cells including staining for SMA to confirm that the cultures were not contaminated with SMC. Endothelial markers appeared to be reduced in endothelium in aneurysms from patients with both TAV and BAV (Figure 3).

**Figure 4:**
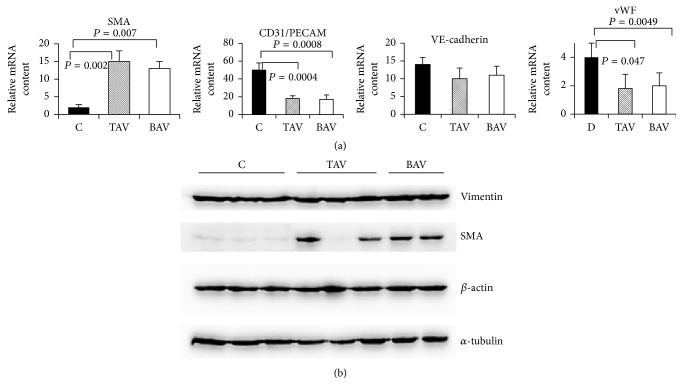
Expression of smooth muscle cell (SMC) markers and endothelial cell markers in endothelial cells from patients with aortic aneurysm with either tricuspid aortic valve (TAV) or bicuspid aortic valve (BAV) and controls (C). mRNA level was determined by qPCR; protein level was determined by Western blot. The diagrams represent the results of densitometry. The bands were normalized by *β*-actin. (a) mRNA and protein level in endothelial cells: controls: *n* = 5; TAV: *n* = 5; BAV: *n* = 5; (b) representative Western blot picture for SMA and vimentin protein level in endothelial cells.

We compared also the mRNA level of SMA, CD31/PECAM, VE-cadherin, and vWF in endothelial cells derived from control aortas and from aneurysms. The expression of SMA mRNA was elevated in both TAV- and BAV-derived endothelial cells (Figure 4). Typical SMA microfilament staining was not observed in our endothelial cultures (Figure 3); thus the elevation of SMA mRNA level was not due to contamination with SMC. Expression of the endothelial markers vWF and CD31/PECAM was substantially decreased in endothelial cells from aneurysms (Figure 4); the level of VE-cadherin mRNA was not changed.

### 3.3. Proliferation and Migration of Cells from Aneurysms

**Figure 5:**
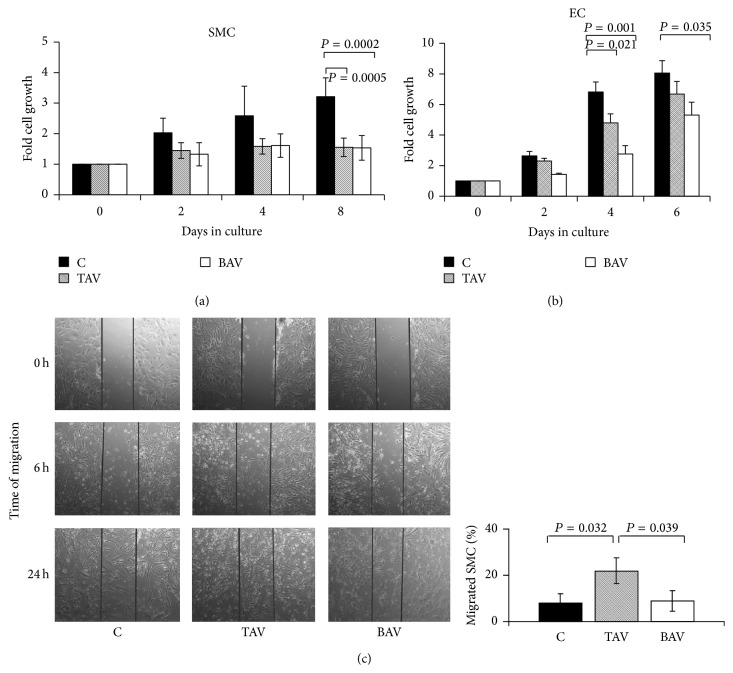
Proliferation and migration characteristics of SMC and endothelial cells from patients with aortic aneurysm with either tricuspid aortic valve (TAV, *n* = 5) or bicuspid aortic valve (BAV, *n* = 5) and controls (C, *n* = 5). The cells were seeded at an equal density and counted each two days for proliferation assay. The migration was estimated via scratch-assay (see Section 2). Migrated cells were counted after 6 h and 24 hours. (a) SMC proliferation. (b) Endothelial proliferation. (c) SMC migration.

Specific degenerative processes associated with reduced cellularity are observed in the aneurysm wall [19]. To evaluate the possible contribution of SMC and endothelial cells to these changes, we compared cell proliferation and migration in healthy donors and patients with aneurysms of the thoracic aorta. SMC proliferation rate (Figure 5(a)) in both BAV and TAV aneurysm was lower compared to healthy donors, but without any difference between the two types of aneurysms. Endothelial cells from aneurysm patients had also lower proliferation than controls, but endothelium from patients with BAV had lower proliferation rate than endothelium from aneurysms of patients with TAV (Figure 5(b)).

SMC migration rate was higher in aneurysm patients with TAV, but not in BAV patients compared to controls (Figures 5(a) and 5(b)).

### 3.4. Apoptosis in Smooth Muscle Cells from Aneurysm Walls

**Figure 6:**
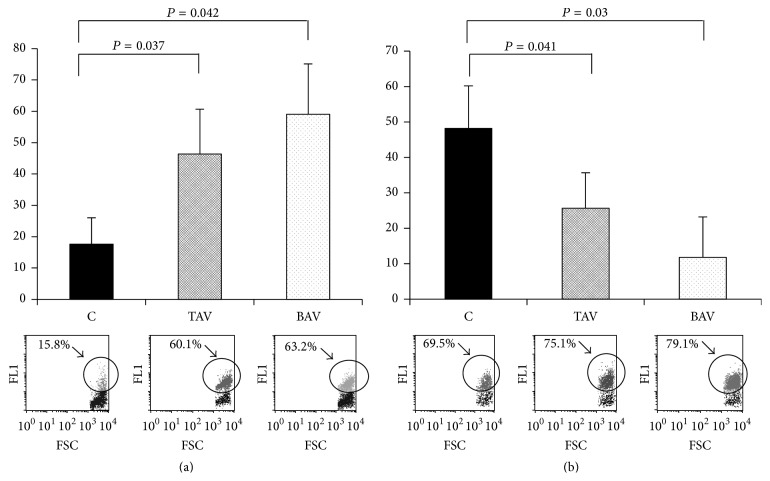
Apoptosis level in cultured SMC from patients with aortic aneurysm with either tricuspid aortic valve (TAV) or bicuspid aortic valve (BAV) and controls (C). (a) The level of “baseline” SMC apoptosis in culture. The diagram shows the percentage of annexin V positive SMC in vitro estimated by flow cytometry. The lower panel shows representative plots from the analysis of live SMC in culture. C: *n* = 5; TAV: n=5; BAV: *n* = 5. (b) Apoptosis induction by H_2_O_2_. The diagram shows the residual between the percentage of annexin V positive cells after H_2_O_2_ treatment and the level of annexin V positive cells in untreated cultures. The lower panel shows representative plots from the analysis of SMC treated with H_2_O_2_. C: *n* = 5; TAV: *n* = 5; BAV: *n* = 5. Arrows mark annexin V positive cells.

Reduced cell number has been shown in aortic tissue of thoracic aortic aneurysm patients [20] and may reflect increased apoptotic level in the vessel wall cells. Indeed, the number of cells that are positive for DNA double strand breaks (an apoptotic marker) is increased in the media of the wall of thoracic aneurysms [20]. Therefore we studied apoptosis in SMC cultures from aneurysm patients and controls (Figure 6(a)). The number of annexin V positive cells was significantly higher in SMC cultures from patients with both BAV and TAV.

Oxidative injury might be a cause of increased wall weakness in both abdominal and thoracic aortic aneurysm [21]. The ability of oxidative stress to cause apoptosis might be altered in SMC from aneurysms. To test this hypothesis H_2_O_2_ was added to SMC. We measured apoptosis as a residual between the percentage of annexin V positive cells after H_2_O_2_ treatment and the amount of annexin V positive cells in normal cultures (“baseline”). H_2_O_2_-induced apoptosis was reduced in SMC from aneurysms compared to SMC from nonaneurysm aortic walls (Figure 6(b)).

### 3.5. Matrix Metalloproteases and Matrix Protein Content

**Figure 7:**
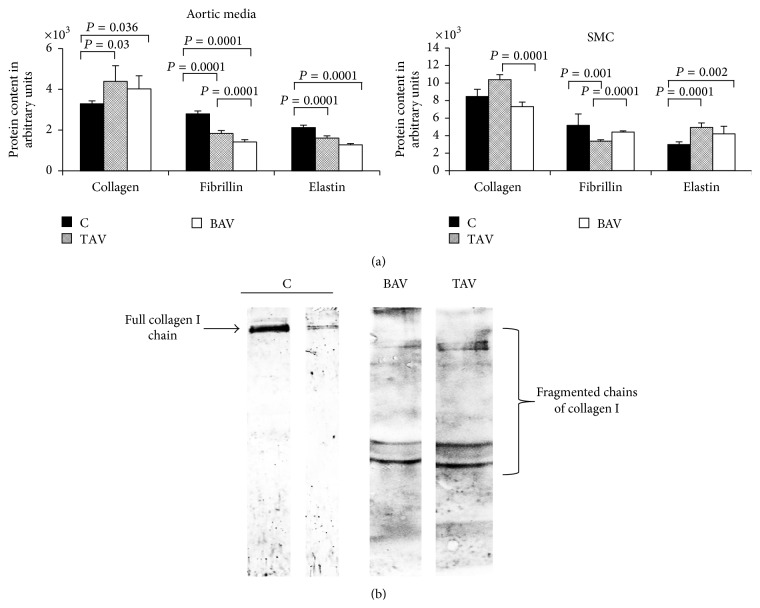
Matrix protein level in SMC and endothelial cells from patients with aortic aneurysm with either tricuspid aortic valve (TAV) or bicuspid aortic valve (BAV) and controls (C) determined by Western blot. (a) Matrix protein level in SMC. The diagrams represent the results of densitometry. The bands were normalized by *β*-actin. C: *n* = 10; TAV: *n* = 13; BAV: *n* = 17. (b) Collagen I protein level in endothelial cell culture media.

To evaluate SMC contribution to extracellular matrix protein synthesis (elastin, fibrillin, and collagen I) in the aortic wall we estimated the protein content in aortic media specimens and in SMC protein extracts (Figure 7). The elastin and fibrillin content was reduced in aortic media from aneurysms in both BAV and TAV patients (Figure 7(a)).

Collagen I content was higher in aortic media from both types of aneurysm patients but was not significantly changed in SMC from aneurysm patients. However, the amount of collagen I was higher in SMC of aneurysms from patients with TAV only (Figure 7(a)).

The culture media from endothelial cells were analyzed for elastin, collagen I, and fibrillin content (Figure 7(b)). Our data show that aortic endothelial cells are capable of synthesizing these proteins and synthesizing fragmented collagen I.

**Figure 8:**
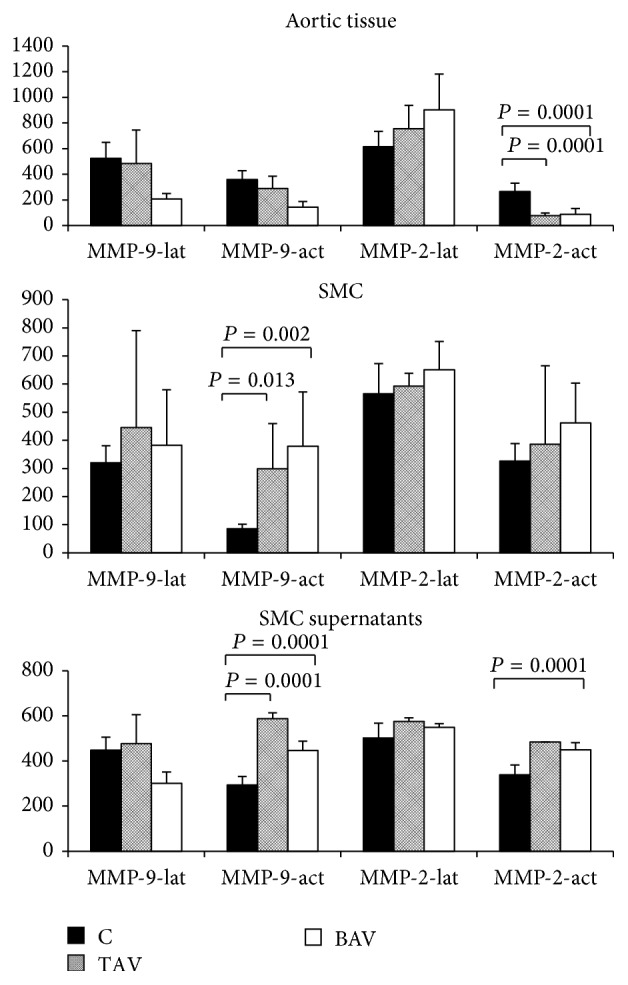
The level of MMP2 and MMP9 activity in SMC from patients with aortic aneurysm with either tricuspid aortic valve (TAV) or bicuspid aortic valve (BAV) and controls (C) determined by different groups of samples determined by zymography. C: *n* = 5; TAV: *n* = 5; BAV: *n* = 5. MMP-2-lat, MMP-9-lat: latent forms of MMP2 and MMP9, correspondingly. MMP-2-act, MMP-9-act: active forms of MMP2 and MMP9, correspondingly.

MMP expression may be increased in thoracic aneurysms [2, 22], but their role in the etiology of aneurysms is not clarified. The exact type of cells in the aortic wall that synthesizes MMPs is still unknown [23]. We did not detect increase in MMP2 or MMP9 activity in total aneurysm medial tissue samples (Figure 8), whereas MMP-9 activity was significantly increased in SMCs and SMC culture media from aneurysm patients with both BAV and TAV.

## 4. Discussion

The functional studies of the cells from TAA patients are still rare. However the ultimate target of any therapy approach is a cell, whose healthy properties are changed in the pathology. That is why, it is important to know not only a set of genes changed at a definite pathology, but also cellular properties that are the cause of a pathology and thus to find a possible tool for a correction. This study aimed to characterize two major aortic cellular populations, SMC and EC, from TAA patients and healthy donors.

In the present study we demonstrated that both SMC and endothelial cells from thoracic aortic aneurysms have impaired functional properties in terms of proliferation, contractile and extracellular protein expression, and apoptosis with significant difference between BAV- and TAV-associated aneurysms. Of most importance, we revealed the primary changes of endothelial cells. Our findings are in agreement with a recent publication describing endothelial dysfunction in BAV patients [11].

SMC from thoracic aneurysms demonstrated decreased expression of key SMC proteins such as SMA and SM22*α* both at mRNA and at protein level. Endothelial cells from thoracic aneurysms also demonstrated downregulation of specific markers and impairment in growth. Our data suggests that the cells from the aneurysm aortic wall are in less differentiated state comparing to normal aortic wall. This observation is well in line with the findings that endothelial cells influence differentiation and functions of underlying SMC [13, 14]. Thus, endothelial changes may contribute to impairment of the aortic wall structure built by SMC.

Cultured SMC from aneurysm patients demonstrated surprisingly high amounts of annexin V positive cells suggesting a high level of apoptosis in the aortic wall of aneurysm patients [3]. This may also be partly a consequence of the cell culture, but nevertheless there were differences between the different groups. Surprisingly the SMCs were relatively resistant to apoptosis induced by H_2_O_2_. This may be an important finding, but we have no good explanation. Philippi and coauthors showed that SMC from aneurysms in BAV patients had the poorest resistance to oxidative stress [10], but they did not show a baseline level of apoptosis in their SMC cultures but counted the viability of the cells under oxidative stress. Apoptosis/proliferation rate is a very important parameter of a cellular population turnover. We suggest that both decreased proliferation rate and increased apoptosis contribute to the loss of cells in the aortic wall in aneurysms. The mechanisms of apoptosis resistance/susceptibility in aortic SMC population may be important in the development of aneurysms.

We compared the content of some extracellular matrix protein in aortic tissue samples, SMC and supernatants from SMC and endothelial cells. The observed results suggest that SMC are not the only cells that synthesize extracellular matrix proteins in the aortic wall. Also endothelial cells are capable of synthesizing extracellular matrix proteins in aneurysms and the extracellular matrix composition is different between aneurysms from patients with TAV and BAV TAA. Finally the integrity of the extracellular matrix in the wall of aneurysms is influenced by changes in the ratio between different proteins. Fibrillin content was different between aneurysms from TAV and BAV patients. Both endothelial cells and SMC from aneurysm patients had synthesis of fragmented collagen I. The complex nature of biosynthesis of extracellular matrix proteins in the aneurysm wall is in agreement with other recent studies [22, 23].

The role of different MMP in thoracic aneurysm pathogenesis has been discussed [22, 23]. There are a number of papers describing mainly the elevated gene expression levels for some MMPs in the aneurysm wall [2, 22–24]. In our study we analyzed the enzyme activity of MMP2 and MMP9. The activity for MMP2 and MMP9 was elevated in SMC and in supernatants from SMC cultures. This is in accordance with previously reported elevated gene expression of MMP2 and MMP9 in the wall of thoracic aneurysms [22, 23].

This study has several important limitations. The study is heterogeneous because from every tissue sample it is not possible to get SMC and endothelial cell cultures; the cells are in culture for limited amount of passages that limits the possibilities to study them. For the endothelial cells it is difficult to get cultured cells and it is not possible to get enough cells from many patients for different kind of analysis. Another limitation is that we have studied only two types of cells whereas the aorta consists of more types of cells including stem cells, macrophages, and fibroblasts. A very important limitation is that gene expression and cell behavior in dilatative aortopathy can be affected by hemodynamics as well as by physical and biochemical environment, other than by their interaction with the genetic background and these factors are absent in cultured cells.

Further research of the cell alterations in the wall of thoracic aneurysms might lead to a deeper understanding of pathogenesis and pathology and thus to find a potential therapeutic tool.

## Supplementary Material

In the supplementary figure 1 we demonstrate the immunohistochemical staining of ascending aortic wall specimens for smooth muscle cell markers (calponin, vimentin, SM22α and αSMA) and for endothelial cell markers (CD31/PECAM, vWF) in healthy donors (control), in the patients with aortic aneurysm and bicuspid aortic valve (BAV), and in the patients with aortic aneurysm and normal tricuspid aortic valve (TAV)

## Figures and Tables

**Table 1:** Clinical characteristics in the study groups.

	TAV (*n* = 13)	BAV (*n* = 22)
Male gender (%)	46	59
Age (years)	71.3 ± 2.53 (range 55–84)^*∗*^	62.1 ± 1.87 (range 42–79)^*∗*†^
Aortic diameter (cm)	5.6 ± 0.18^*∗*^	5.9 ± 0.16^*∗*^
Aortic CSA/h (cm^2^/m)	6.6 ± 0.6^*∗*^	7.6 ± 0.6^*∗*^
Peak valve gradient (mmHg)	83 ± 9	86 ± 11
Mean valve gradient (mmHg)	55 ± 7	59 ± 9
Aortic valve area index (cm^2^/m^2^)	0.39 ± 0.02	0.38 ± 0.02
Hypertension (%)	84^*∗*^	81^*∗*^
Medication		
Angiotensin receptor blockers (%)	38^*∗*^	18
Statins (%)	0	41^*∗*†^
Aspirin (%)	31	14

Values are means ± SEM. ^*∗*^ *P* < 0.05 compared with donors; ^†^ *P*<0.05 compared with TAV; CSA/h, ascending aortic cross-sectional area to patient height ratio.
