# Supplementary figures and images for: Phenotypic and Functional Changes of Endothelial and Smooth Muscle Cells in Thoracic Aortic Aneurysms
Source: Int J Vasc Med. 2016 Jan 19;2016:3107879. doi: 10.1155/2016/3107879 (PMC4745582; doi:10.1155/2016/3107879)

A

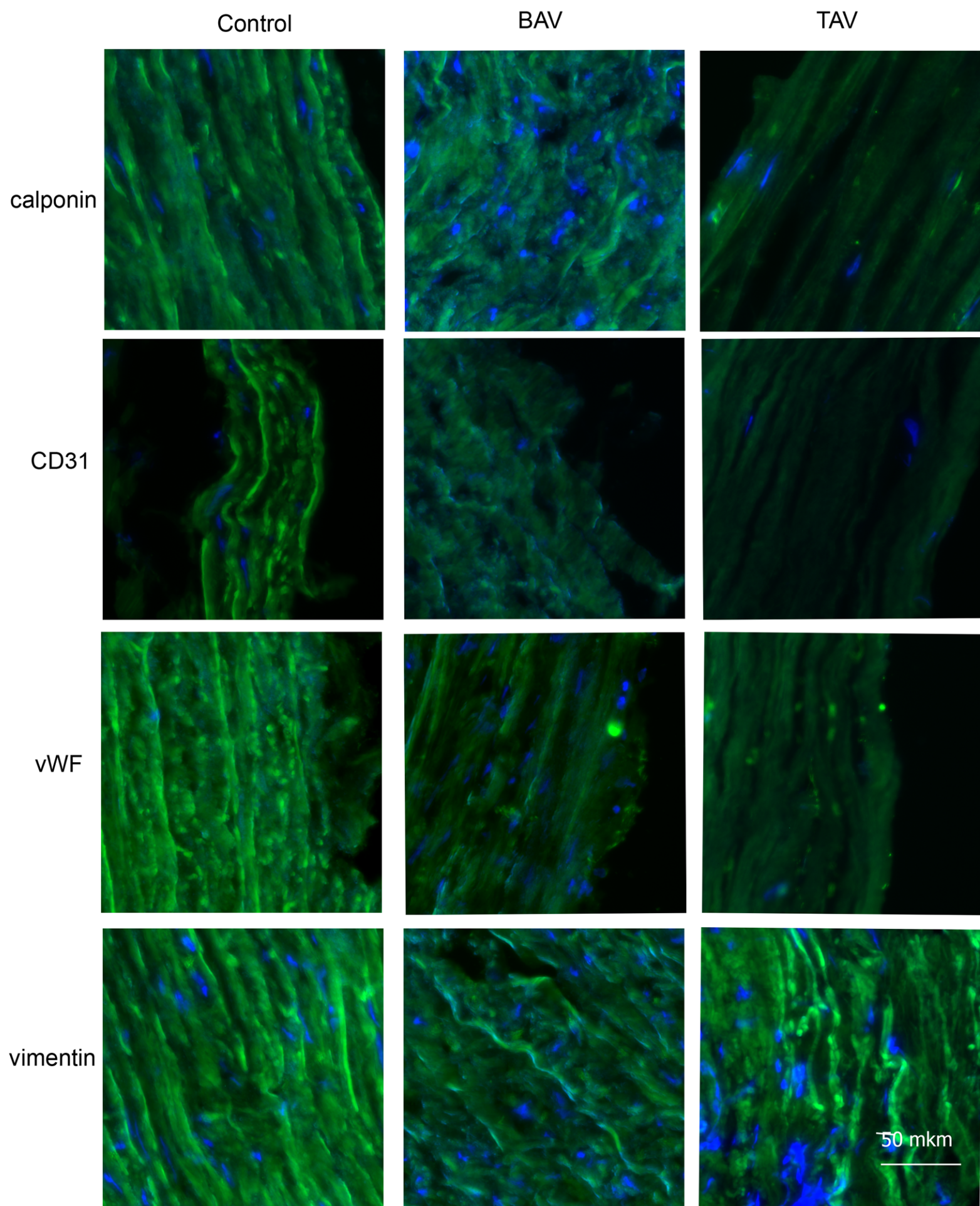

Supplement: Supplementary file 1 — In the supplementary figure 1 we demonstrate the immunohistochemical staining of ascending aortic wall specimens for smooth muscle cell markers (calponin, vimentin, SM22α and αSMA) and for endothelial cell markers (CD31/PECAM, vWF) in healthy donors (control), in the patients with aortic aneurysm and bicuspid aortic valve (BAV), and in the patients with aortic aneurysm and normal tricuspid aortic valve (TAV) [file 3107879.f1.zip › Suppl_figure1_A_IJVM_1521697.pdf]

A

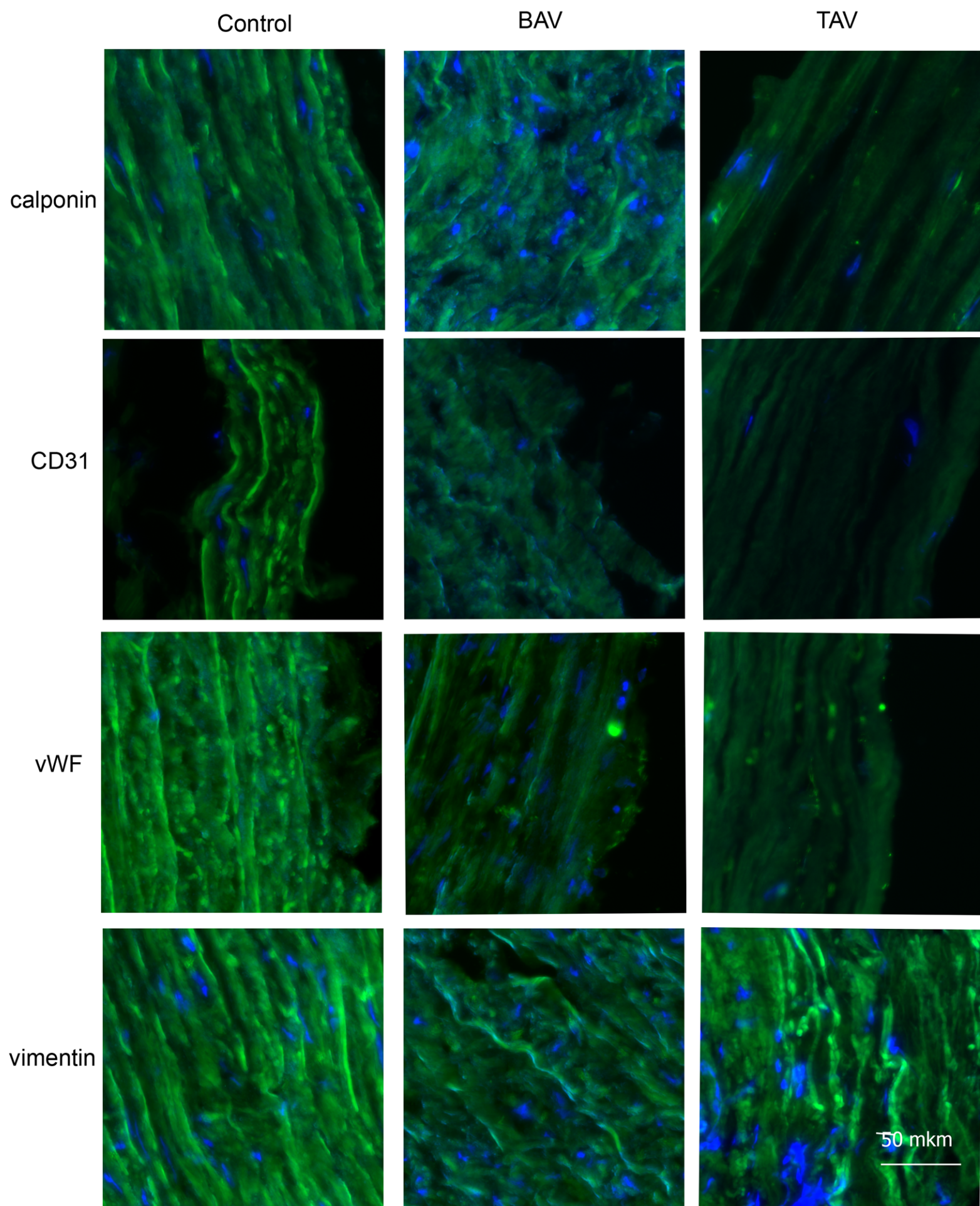

Supplement: Supplementary file 1 — In the supplementary figure 1 we demonstrate the immunohistochemical staining of ascending aortic wall specimens for smooth muscle cell markers (calponin, vimentin, SM22α and αSMA) and for endothelial cell markers (CD31/PECAM, vWF) in healthy donors (control), in the patients with aortic aneurysm and bicuspid aortic valve (BAV), and in the patients with aortic aneurysm and normal tricuspid aortic valve (TAV) [file 3107879.f1.zip › Suppl_figure1_A_IJVM_1521707.pdf]

B

control

BAV

TAV

SM22

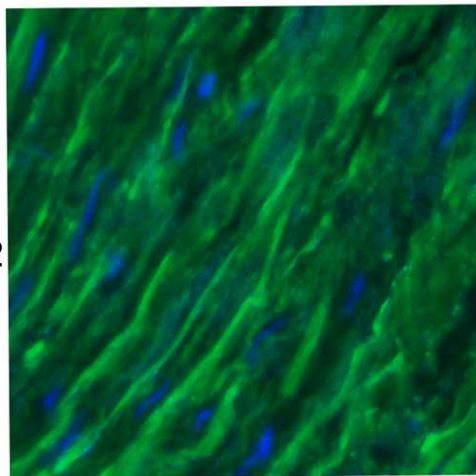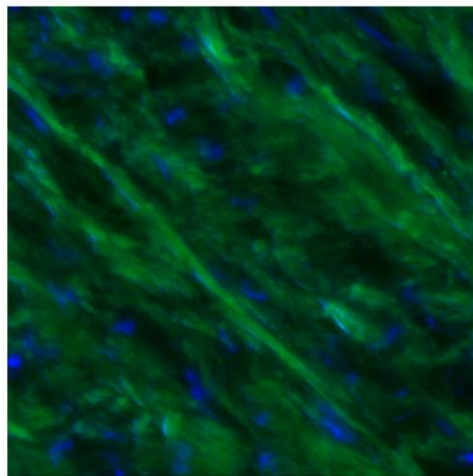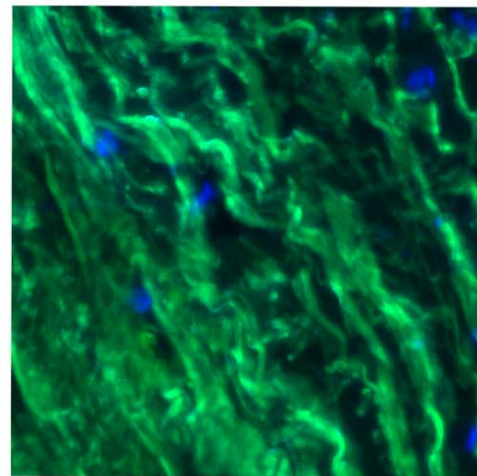

SMA

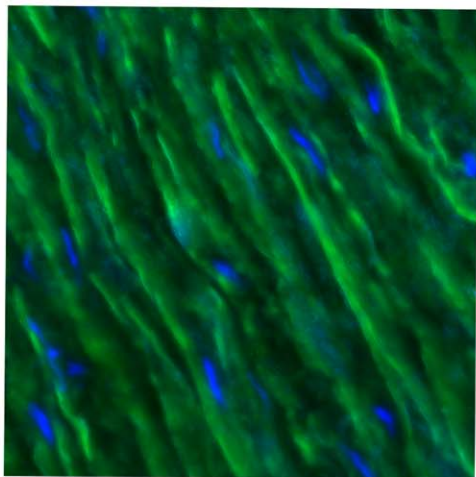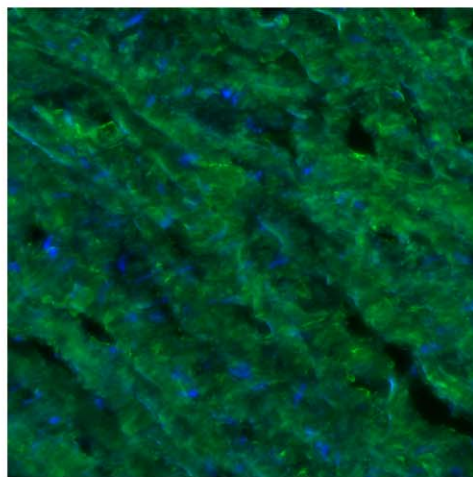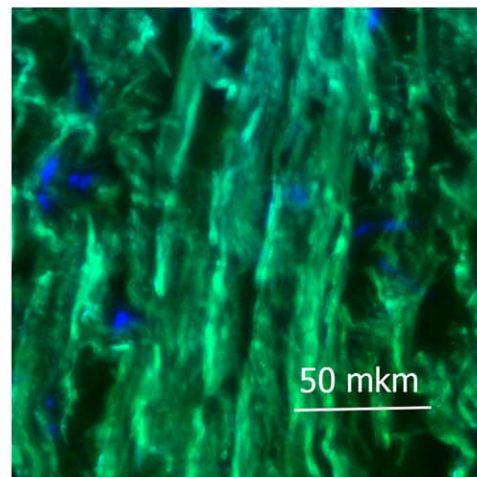

Supplement: Supplementary file 1 — In the supplementary figure 1 we demonstrate the immunohistochemical staining of ascending aortic wall specimens for smooth muscle cell markers (calponin, vimentin, SM22α and αSMA) and for endothelial cell markers (CD31/PECAM, vWF) in healthy donors (control), in the patients with aortic aneurysm and bicuspid aortic valve (BAV), and in the patients with aortic aneurysm and normal tricuspid aortic valve (TAV) [file 3107879.f1.zip › Suppl_figure1_A_IJVM_1521708.pdf]

B

control

BAV

TAV

SM22

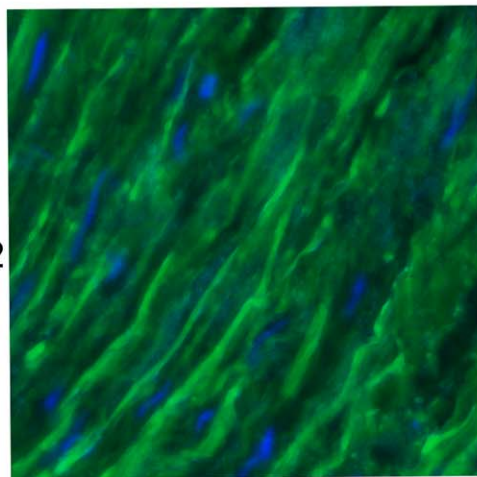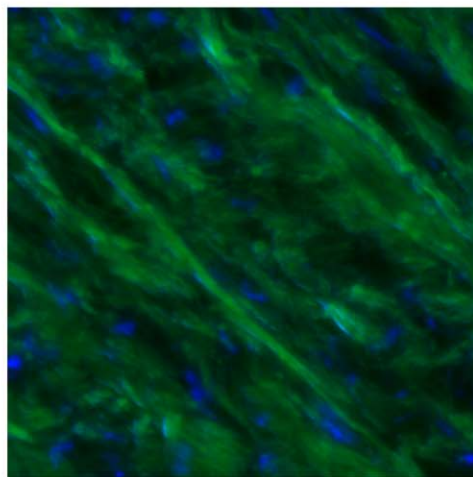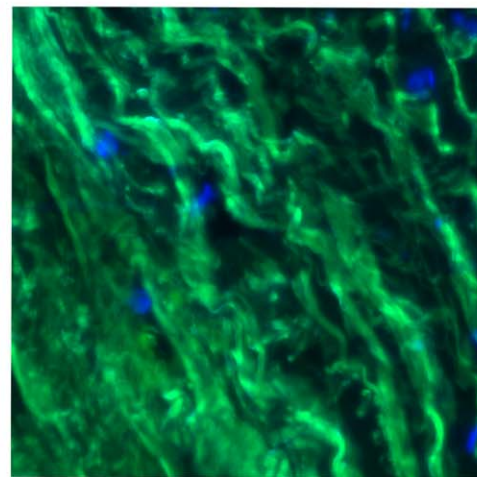

SMA

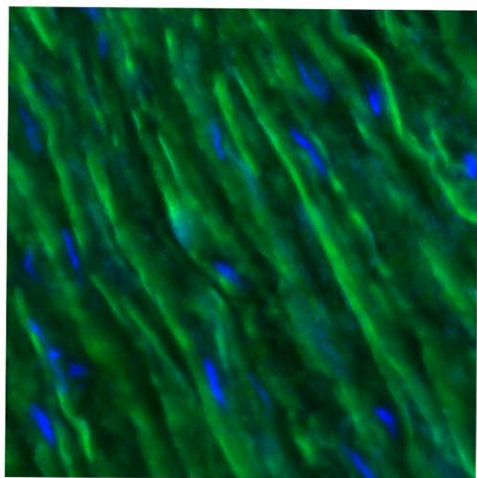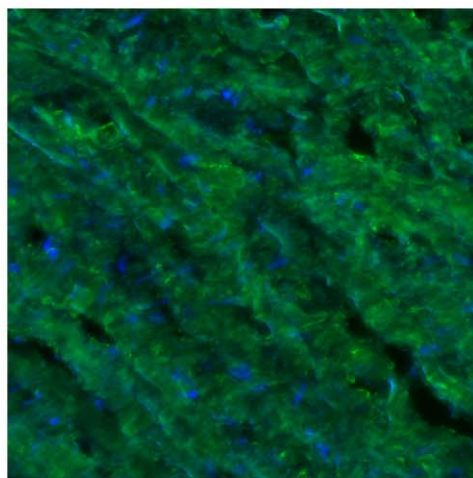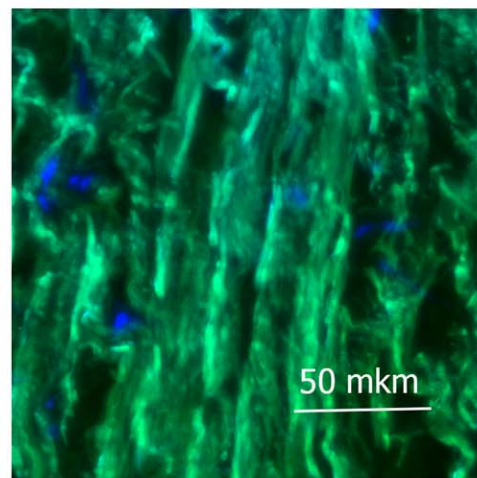

Supplement: Supplementary file 1 — In the supplementary figure 1 we demonstrate the immunohistochemical staining of ascending aortic wall specimens for smooth muscle cell markers (calponin, vimentin, SM22α and αSMA) and for endothelial cell markers (CD31/PECAM, vWF) in healthy donors (control), in the patients with aortic aneurysm and bicuspid aortic valve (BAV), and in the patients with aortic aneurysm and normal tricuspid aortic valve (TAV) [file 3107879.f1.zip › Suppl_figure1_A_IJVM_1521798.pdf]
